# Functional Mapping of the Human Visual Cortex with Intravoxel Incoherent Motion MRI

**DOI:** 10.1371/journal.pone.0117706

**Published:** 2015-02-03

**Authors:** Christian Federau, Kieran O’Brien, Adrien Birbaumer, Reto Meuli, Patric Hagmann, Philippe Maeder

**Affiliations:** 1 Department of Diagnostic and Interventional Radiology, Centre Hospitalier Universitaire Vaudois (CHUV) and University of Lausanne, Lausanne, Switzerland; 2 Center for Biomedical Imaging (CIBM), University of Geneva, Geneva, Switzerland; University of Michigan, UNITED STATES

## Abstract

Functional imaging with intravoxel incoherent motion (IVIM) magnetic resonance imaging (MRI) is demonstrated. Images were acquired at 3 Tesla using a standard Stejskal-Tanner diffusion-weighted echo-planar imaging sequence with multiple b-values. Cerebro-spinal fluid signal, which is highly incoherent, was suppressed with an inversion recovery preparation pulse. IVIM microvascular perfusion parameters were calculated according to a two-compartment (vascular and non-vascular) diffusion model. The results obtained in 8 healthy human volunteers during visual stimulation are presented. The IVIM blood flow related parameter fD* increased 170% during stimulation in the visual cortex, and 70% in the underlying white matter.

## Introduction

Focal brain neural activity increases local perfusion through neurovascular coupling [1]. Vascular-based brain imaging techniques, such as positron emission tomography (PET) [2] and functional magnetic resonance imaging (fMRI), first with susceptibility-contrast MRI [3] and currently using the blood oxygenation level-dependent (BOLD) effect [4–6], have provided images of the “brain in action”, demonstrating patterns of brain activity in relation to behavior or somatosensory input. BOLD fMRI is based on the variation of the blood water T_2_/T_2_* signal, which depends on the paramagnetic deoxyhemoglobin content [7, 8]. This method is robust but faces challenges due to the non-trivial signal dependence on several parameters (cerebral blood flow, cerebral blood volume, and blood oxygenation) [9–14], while the spatial resolution is limited due to veins draining the sites of activation [15]. An MRI method capable of measuring variation in microvascular blood flow during neuronal activation, independently of blood oxygenation, is therefore of interest.

Capillary network reactivity to somatosensory stimulation has been investigated in rats [16, 17], and individual capillary increase in red blood cell velocity and flow has been demonstrated with two-photon microscopy during the activation of the olfactory bulb [18] and neocortex [19]. In humans, microvascular perfusion measurement is possible with intravoxel incoherent motion (IVIM) magnetic resonance imaging MRI [20, 21]. This method is based on the natural dependence of the nuclear magnetic resonance (NMR)/MRI signal on nuclei motions [22, 23]; this dependence can be accentuated by the use of pulsed gradients [24]. The vasculature is assumed to be sufficiently dense and random so that blood movements present statistical, diffusive properties, and a pseudo-diffusion coefficient D* can be introduced. This coefficient is calculated using a bi-compartmental (vascular and non-vascular) model [20], with the second compartment undergoing thermal diffusion D. Further perfusion parameters can be derived, namely, the perfusion fraction f, and the flow-related parameter fD*, which consists of the product of f and D* [21]. The IVIM method of measuring human brain perfusion has been recently validated, showing dependence on hypercapnia-induced vasodilatation [25].

Diffusion gradients have been used for various purposes in fMRI [26], but usually not for deriving specifically microvascular perfusion parameters using the IVIM model. An early use was to modulate the BOLD signal [27] to try to localize it and to increase its spatial resolution by suppressing the signal from flowing blood [28–31]. Diffusion gradients have also been used to directly measure changes in the apparent diffusion coefficient (ADC) during fMRI, using a single-compartment model. At low b-values, functional ADC measurements have shown potential for both increased spatial [32, 33] and temporal resolution [34]. At high b-values, an effect could also be measured, in the form of a temporary decrease in ADC that has been measured in the human visual cortex during stimulation [35], and was found to be significantly faster than the BOLD response [36, 37]. This was interpreted as a direct measure of cell swelling during neural firing, which could represent a more direct and accurate measurement of neuronal activity than hemodynamic-based contrast. The exact nature of this signal remains controversial though, as it has been suspected to arise from vascular and susceptibility effects [38] as well as from partial volume effect with cerebro-spinal fluid (CSF) [39]. Indeed, a decrease in CSF volume during brain activation, as well as during hypercapnia, has been observed with various methods [40–43], and has been suggested as a possible confounding factor in ADC-fMRI [39]. This effect has also been observed with IVIM during hypercapnia [25].

In this paper, we investigated the feasibility of measuring variation of local microvascular brain perfusion parameters f, D*, and fD* in human volunteers during visual stimulation, as derived from the bi-compartmental IVIM model, using a diffusion-weighted inversion-recovery sequence to suppress the possibly confounding CSF movements.

## Material and Methods

### Subjects

This study was approved by the local ethics committee at the University Hospital in Lausanne (Commission cantonale (VD) d’éthique de la recherche sur l’être humain). Informed written consent was obtained from all participants. Imaging was performed on 8 healthy subjects without known history of disease (4 men and 4 women, mean age 25), all over 18 years of age, from April 2012 to July 2012. No subject had to be excluded from this study.

### Visual Stimulation

An LCD projector equipped with a photographic zoom lens and with a refresh rate of 75 Hz displayed the stimuli on a translucent screen positioned in the back part of the bore. Subjects viewed stimuli through a custom-made inclined mirror positioned above their eyes, and had a field of view of ± 20° horizontally and ± 11° vertically. Subjects were asked to look at a fixation point in the middle of the screen. Total distance eye to screen was 1 m. Head motion was kept to a minimum using a vacuum bag.

Subjects were presented with the following sequence: visual stimulus blocks alternating with black screen blocks, always starting with the stimulus block. The blocks were 9 min 32 sec each. The visual stimulus block consisted of a red and black checkerboard (12 squares horizontally and 9 vertically, each measuring 2.5 cm^2^ on the screen), blinking with a frequency of 8 Hz (where “blinking” means that each individual square alternated between being red and being black).

We acquired an IVIM sequence for each block presented to the subject. 5 (in some cases 3) IVIM sequences were acquired for each subject. The visual stimulus or the black screen was started 20 s before the acquisition for equilibrium; so, each IVIM acquisition was 9 min 12 sec long.

### Images Acquisitions

Data were acquired on a 3 Tesla MR scanner (Trio, Siemens, Erlangen, Germany) using a 32-multichannel receiver head coil. For the purpose of localization, the acquisition was started by a T1-weighted high-resolution (1 mm isotropic) MPRAGE sequence (TR = 2.3 s, TE = 3 ms, TI = 900 ms, flip angle = 9°, field of view = 256 x 240 mm^2^, matrix size = 256 x 240, slice thickness = 1.2 mm, Bandwidth 238 Hz/pixel), followed by a standard functional visual experiment, which consisted of a BOLD sensitive gradient echo EPI sequence (TR = 4 s, TE = 30 ms, flip angle = 90°, field of view = 192 x 192 mm^2^, matrix size = 64 x 64, slice thickness = 3 mm, Bandwidth 2232 Hz/pixel). 60 images were acquired in total, alternating 10 acquisitions during stimulation and 10 during baseline. Single participant analysis was performed using the General Linear Model according to our specific block design experiment. The resulting computed t-maps were then used to identify the visual cortex, and a single IVIM slice was placed in a strict transverse plane on the calcarine fissure.

### IVIM Imaging Parameters

Data were acquired using a Stejskal-Tanner diffusion-weighted adiabatic inversion-recovery (TI = 2660 ms) spin echo sequence [24] and echo planar read-out [44]. A long repetition time of 12 s was applied to ensure complete recovery of each tissue. A single axial brain slice of 7 mm thickness was acquired with an in-plane resolution of 1.2 x 1.2 mm^2^, using a field of view of 256 x 256 mm^2^ and a matrix of 210 x 210. TE was 92 ms. Parallel imaging with an acceleration factor of 2, and 75% partial Fourier encoding in phase direction was applied. Receiver bandwidth was 1134 Hz/pixel. Fat was suppressed with a frequency selective saturation pulse. Images were acquired at multiple b-values (0, 10, 20, 40, 80, 110, 140, 170, 200, 300, 400, 500, 600, 700, 800, 900 s/mm^2^), in 3 orthogonal directions, from which the traces were calculated, which were then used for model fitting. Images were acquired only once for each b-value and direction, and only once for b = 0. Eddy current induced spatial distortions were corrected using the vendor's software.

### Region of Interest (ROI) Definition and Segmentation

For quantitative analysis, a visual brain region and a non-visual brain region were obtained by thresholding the t-maps of the BOLD signal. Those two ROIs were further segmented in gray (GM) and white matter (WM) with the help of probability maps constructed from a MPRAGE sequence using the segment function of the SPM framework (http://www.fil.ion.ucl.ac.uk/spm) for Matlab (Mathworks, Natick, MA, USA). Those maps were registered to the IVIM space using 3D Slicer (http://www.slicer.org). The segmentation maps were finally corrected manually on a voxel-by-voxel basis, using a homemade Matlab program. Regions with significant susceptibility artifacts (petrous bone, frontal sinuses) were excluded. The 4 obtained ROIs (GM and WM in the visual and non-visual brain, respectively) are presented in S1 Fig, supplementary material.

### Image Processing and Analysis

We used the double exponential model proposed by le Bihan et al [20]

$$\frac{S(b)}{S_{0}}=f\cdot e^{-bD^{*}}+(1-f)\cdot e^{-bD}$$

where *S*(*b*) and *S*_0_ represent the signal obtained at a given b-value and with no gradient applied, respectively. Data were fitted in two steps: first, the curve was fitted for b > 200 s/mm^2^ for the single parameter D, followed by a fit for f and D* over all values of b, while keeping D constant, using the Levenberg-Marquardt algorithm [45] implemented with standard Matlab functions. The curve fitting in the parametric maps was done on a voxel-by-voxel basis, while for quantitative analysis, it was done after averaging the signal of the ROI for each b-value. The later was done to avoid choosing an arbitrary cut-off for misfitted points, which might influence the results.

### Parameter Fitting Simulation

The quality of the fitting procedure was evaluated with two simulations, the first assessing the quality of the fit as a function of signal-to-noise (SNR), and the second, the quality of the fit as function of f and D* under the measured intravoxel SNR of the current experiment.

### First simulation: fitting quality as a function of SNR

In the first simulation, an SNR-dependent Gaussian random noise term was added at each b-value to the ideal signal corresponding to f = 4%, D = 0.7·10^-3^ mm^2^·s^-1^, and D* = 17·10^-3^ mm^2^·s^-1^, after which the fitting was performed. Those numerical values were obtained from the experimental values of the gray matter at baseline (Table 1; the values were rounded for simplicity). This was repeated 10’000 times at each SNR ranging from 10 to 400.

**Table 1:** Quantitative measurement of IVIM parameters in the visual and the non-visual brain.

	Gray Matter				White Matter				p-value Gray—White Matter	
Visual Brain	Baseline	Stimulation	Variation	p-value	Baseline	Stimulation	Variation	p-value	Baseline	Stimulation
fD*	0.59 ± 0.32	1.61 ± 0.96	+170%	0.01	0.37 ± 0.11	0.63 ± 0.31	+70%	0.01	0.02	0.005
D*	17.27 ± 10.17	30.50 ± 18.35	+77%	0.048	9.58 ± 4.82	13.40 ± 6.18	+40%	0.0003	0.02	0.01
F	3.55 ± 0.59	5.34 ± 0.81	+50%	0.0001	4.17 ± 0.98	4.69 ± 0.45	+12%	0.08	0.057	0.005
D	0.713 ± 0.017	0.711 ± 0.005	+0%	0.36	0.723 ± 0.019	0.719 ± 0.017	-1%	0.30	0.059	0.09
Non-Visual Brain	Baseline	Stimulation	Variation	p-value	Baseline	Stimulation	Variation	p-value	Baseline	Stimulation
fD*	0.70 ± 0.40	0.58 ± 0.15	-17%	0.22	0.57 ± 0.57	0.44 ± 0.28	-23%	0.13	0.12	0.04
D*	16.97 ± 11.29	17.91 ± 9.45	+6%	0.42	15.13 ± 20.81	9.65 ± 4.54	-34%	0.19	0.32	0.03
F	4.67 ± 3.01	3.66 ± 1.27	-21%	0.10	4.54 ± 1.57	4.39 ± 0.82	-3%	0.39	0.41	0.04
D	0.724 ± 0.049	0.739 ± 0.016	+2%	0.20	0.714 ± 0.049	0.713 ± 0.020	+0%	0.46	0.11	0.01

The IVIM perfusion parameters D* [10^-3^ mm^2^·s^-1^], fD* [10^-3^ mm^2^·s^-1^] and f [%], as well as the diffusion coefficient D [10^-3^ mm^2^·s^-1^], obtained in the white and gray matter of a region of interest in the visual cortex and in the rest of a full axial slice excluding the occipital lobe.

### Second simulation: fitting quality as a function of experimental, b-value-dependent SNR

The SNR of all baseline images was first measured in the current experimental setting in the whole parenchyma, excluding regions of obvious artifacts. It was calculated for each voxel as a function of b as the deviation of the single measurements from their averaged value. The corresponding Gaussian random noise term was then added at each value of b to the ideal signal corresponding first to D = 0.7·10^-3^ mm^2^·s^-1^, D* = 17·10^-3^ mm^2^·s^-1^ and f ranging from 0.2% to 20% in steps of 0.2%, and second to D = 0.7·10^-3^ mm^2^·s^-1^, f = 4%, and D* ranging from 0.8·10^-3^ mm^2^·s^-1^ to 30·10^-3^ mm^2^·s^-1^ in steps of 0.2·10^-3^ mm^2^·s^-1^. The fitting procedure was then performed. At each point, the simulation was repeated 30'000 times.

### Conversion to standard perfusion units

IVIM parameters have been converted to standard perfusion units by adapting the formulas from [21] to the units used in this report:

$$CBV\;\left[\frac{ml}{100ml}\right]=\lambda_{H_{2}O}\cdot f\cdot 100=0.78\;\cdot \;f\;[\%],$$

$$CBF\;\left[\frac{ml}{100ml\;min}\right]=60\cdot \frac{6\lambda_{H_{2}O}}{L<l>}\cdot f{\cdot 100\cdot D}^{*}\;=\;130\cdot fD^{*}\;[10^{-3}{\;mm}^{2}s^{-1}],$$

with the MRI visible water content $\lambda_{H_{2}O}\;=\;0.78$, the total capillary length *L* = 2 *mm*, and the mean capillary segment length <l>=0.108mm, as used in [21] and also [46].

Statistical Analysis

Paired, single-tailed Student’s T-test was performed with Excel (Microsoft, Redmont, Wa, USA). Statistical significance was set to p < 0.05.

## Results

### Simulation Results

**Fig 1:**
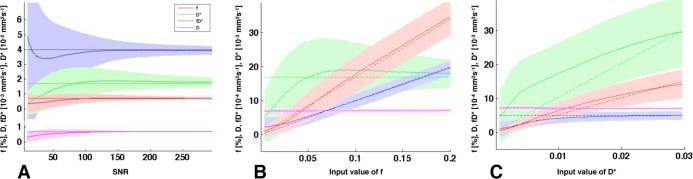
Fit Simulation. (**A**) Simulation fitting result (mean (full line) ± standard deviation (area)) compared to ideal result (dashed line) as a function of SNR, for the perfusion fraction f, the pseudo-diffusion coefficient D*, the flow-related coefficient fD*, and the diffusion coefficient D. For better visualization, the plot of D was separated from the rest of the plot. The fitting results converge toward the ideal value between SNR 50 and 100. (**B-C**) Simulation results of the fitting method (full line) compared to ideal result (dashed line) as a function of the variables f and D*, including experimental noise. Interestingly, in all three simulations, the standard deviation of the fitting of fD* is generally smaller than the one of f and D*.

In the first simulation, the values obtained after fitting converged asymptotically with increasing SNR, reaching the correct value for SNR between 50 and 100 (Fig. 1A). SNR in our experiment was measured to decrease as a function of b (0–900 s/mm^2^) from 107.4 to 34.2. Including this in the second simulation, the quality of the fitting procedure of f, D*, fD*, and D as function of f and D* is shown in Fig. 1 B-C. While the fitting of D was much better than the evaluation of the IVIM perfusion parameters (f, D*, fD*), the quality was found to be acceptable at the current experiment values of f (between 0.035 and 0.053) and D* (between 9.58 and 30.5·10^-3^ mm^2^/s). Interestingly, fD* was found to be more precise than the fit of f and of D* in all three simulations.

### IVIM Functional Imaging Experiment

**Fig 2:**
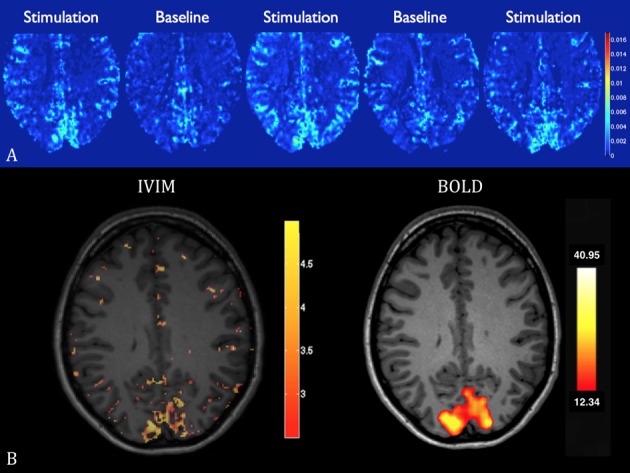
IVIM flow maps. (**A**) In-plane high-resolution (1.2x1.2 mm) maps of the blood flow related IVIM parameter fD*, in 5 consecutive measurements in a single volunteer. An increase in perfusion in the activated primary visual cortex is observed during stimulation compared to baseline. Scale of the colorbar: mm^2^·s^-1^. (**B**) Corresponding IVIM subtraction maps and the corresponding BOLD statistical t-map. Scale of the colorbar: 10^-3^ mm^2^·s^-1^ (IVIM) and t-value (BOLD).

**Fig 3:**
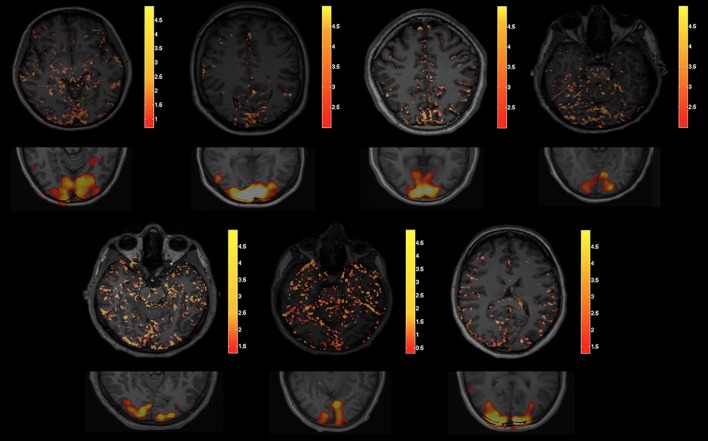
IVIM flow subtraction maps. Maps of the difference in blood flow related IVIM parameter fD*, in all volunteers (except the one already shown in Fig. 2), as obtained by subtracting the averaged flow maps obtained under baseline to the averaged maps obtained under visual stimulation. Scale of the colorbar: 10^-3^ mm^2^·s^-1^. The corresponding BOLD statistical t-map is given below each IVIM subtraction map.

Qualitatively, an increase in flow was observed in the visual cortex on single measurement parametric flow maps fD* during stimulation (Fig. 2, and S2 Fig, supplementary material). Image quality improved after averaging (pixel-wise, after fitting, S3 Fig, supplementary material). Subtraction maps showed an increase in fD* in the primary visual cortex of all volunteers (Fig. 3). An increase in perfusion fraction f and pseudo-diffusion coefficient D* was also visible in the visual cortex, while no variation in diffusion coefficient D was noted (S2 Fig, supplementary material).

Quantitatively, a statistically significant increase of all 3 IVIM perfusion parameters f, D*, and fD* was observed after stimulation in the GM of the visual cortex (50%, p = 0.0001; 77%, p = 0.048; and 170%, p = 0.01, respectively) while a less marked but similar effect was also observed in the visual subcortical WM (12%, p = 0.08; 40%, p = 0.0003; and 70%, p = 0.01 respectively) (Table 1). A trend to a slight decrease of around 20% (p > 0.05 for all variables) in all but one of the IVIM perfusion parameters (Table 1) was observed in the rest of the brain excluding the occipital region. The results in standard perfusion units CBF (cerebral blood flow) and CBV (cerebral blood volume) are presented in Table 2.

**Table 2:** Cerebral blood volume and flow, as derived from the IVIM parameters.

	Gray Matter		White Matter	
Visual Brain	Baseline	Stimulation	Baseline	Stimulation
CBF	76.7 ± 41.6	209.3 ± 124.8	48.1 ± 14.3	81.9 ± 40.3
CBV	2.77 ± 0.46	4.16 ± 0.63	3.25 ± 0.76	3.85 ± 0.35
Non-Visual Brain	Baseline	Stimulation	Baseline	Stimulation
CBF	91.0 ± 52.0	75.4 ± 19.5	74.1 ± 74.1	57.2 ± 36.4
CBV	3.64 ± 2.34	2.85 ± 0.99	3.54 ± 1.22	3.42 ± 1.22

Cerebral blood volume CBV [ml/100ml] and cerebral blood flow CBF [ml/100ml/min], as calculated from the corresponding IVIM perfusion parameters from Table 1. Percentage variations and p-values stay the same and are not reproduced.

## Discussion

### This study demonstrates functional imaging with IVIM MRI in the human visual cortex, as well as in the underlying white matter

This is of interest, because the method has been shown to be quantitative [25], and of microvascular origin [20], and might therefore be seen as a new tool with the potential of sorting respective effects of blood flow and blood oxygenation of the currently used BOLD fMRI technique. The increase of 170% in blood flow is higher than, for example, the 40% increase reported using ASL and PET [47], though similar changes have also been reported, for example 140% CBF increase at 10 Hz stimulation for 60 s, using laser Doppler flowmetry in rats [48]. While the effect was stronger in the cortex, the increase in the WM is noteworthy, because WM BOLD functional activation has remained controversial. Some authors believe it to be undetectable [49], while studies reporting BOLD signal measurement in WM regions are getting more numerous [50–53]. Another interesting finding is the observed trend toward a slight decrease in the IVIM perfusion parameters in the brain excluding the occipital region, which may be artifactual, but may also be related to a reduction in the brain’s baseline default mode activity during specific tasks [54]. Finally, the observed stability of D, using a b_max_ of 900 s/mm^2^, conflicts with previous reports, though there higher b-values were used, such as b_max_ = 1443 s/mm^2^ [35] or b_max_ = 1800 s/mm^2^ [36], and needs further investigation.

Perfusion measurement with IVIM remains an area of active research. In this context, the measured CBV reported here, ranging at rest from 2.77 ± 0.46 to 3.64 ± 2.34 ml/100 ml, is slightly lower but consistent with the CBV of 3.8 ± 0.7 ml/100 ml measured with positron emission tomography [55]. Furthermore, the measured CBF, ranging at rest from 48.1 ± 14.3 to 91.0 ± 75.4 ml/100 ml/min, is of the magnitude order of the generally accepted 50 ml/100 ml/min [56]. Finally, a statistically significant difference was observed in this report between GM and WM for most measured fD*.

There are various sources of possible inaccuracies of the IVIM perfusion parameters as derived in this study. Interestingly, we found fD* to be more precise than the fit of f and of D* in all three simulations, as it seems that the error associated with f and the error associated with D* compensate for each other to some degree. Also, the quality of the fit increased with increased SNR, f, and D* for all perfusion parameters. The corollary is that the quality of the fit is better in regions of increased perfusion (such as the activated regions), which is in the current case the region of interest. Another possible source of inaccuracy is that the different relaxation rates of the various compartments under inversion recovery were not taken into account; however, this allowed us to keep the model simple. Changes in the homogeneity of local magnetic fields due to changes in concentrations of diamagnetic oxyhemoglobin and paramagnetic deoxyhemoglobin could have an effect on the diffusion-weighted signal. Furthermore, it is also well known that T2 of blood is dependent on the oxygenation state of hemoglobin [7], which critically depends on the integrity of the erythrocytes. Therefore, including the relative changes of the dependence on oxygenation of T2 of blood in comparison to the brain parenchyma could improve the accuracy of the IVIM perfusion quantification, but at the cost of increasing the model’s complexity. We used a Gaussian diffusion model to describe the signal decay between b = 200 and 900 s/mm^2^. While more complicated models, such as bi-exponential, or polynomial/kurtosis models exist that better capture the signal decay at high b-values, this would have increased the model’s complexity. Further, changes in inflow/outflow effects (of both CSF and blood) should be considered. The conversion to standard perfusion units CBV and CBF must be understood as a rough estimate, as it depends on the complex and difficult-to-retrieve microvascular topology, which varies greatly in various regions of the brain, for example between white and gray matter, or even between the various layers of the cortex [57]. The MR-visible water content in human brain might also be higher than the value used here [58]. Lastly, the acquisition was limited, on purpose, to single-slice acquisition, to keep the experiment as simple as possible and to exclude any possible multi-slice effects on CSF suppression or blood magnetization, as those components move in the volume in a non-trivial way and might encounter several inversion pulses during the acquisition.

In conclusion, IVIM fMRI can be seen as a new tool for quantitative mapping of microvascular perfusion changes during functional brain activation.

## Supporting Information

S1 Fig Gray and white matter segmentation of visual and non-visual brain. (A) Region of interest of the visual brain, as obtained by thresholding the t-map of the BOLD experiment, further segmented in gray and white matter, and coregistered to the b0 map of the IVIM sequence, and (B) of the non-visual brain, respectively.
(TIF)

S2 Fig Single measurement IVIM parameter maps. (A) Maps of the perfusion fraction f, (B) pseudo-diffusion coefficient D*, (C) flow related coefficient fD*, and (D) diffusion coefficient D, in all 5 (respectively 3) consecutive measurements for all 8 volunteers, during visual stimulation and rest (baseline).
(TIF)

S3 Fig Averaged IVIM flow maps. Maps of the blood flow related IVIM parameter fD*, in 8 volunteers, as obtained by averaging the maps obtained under visual stimulation and baseline. Scale of the colorbar: 10^-3^ mm^2^·s^-1^.
(TIF)
