# Supplementary figures and images for: Functional Mapping of the Human Visual Cortex with Intravoxel Incoherent Motion MRI
Source: PLoS One. 2015 Feb 3;10(2):e0117706. doi: 10.1371/journal.pone.0117706 (PMC4315413; doi:10.1371/journal.pone.0117706)

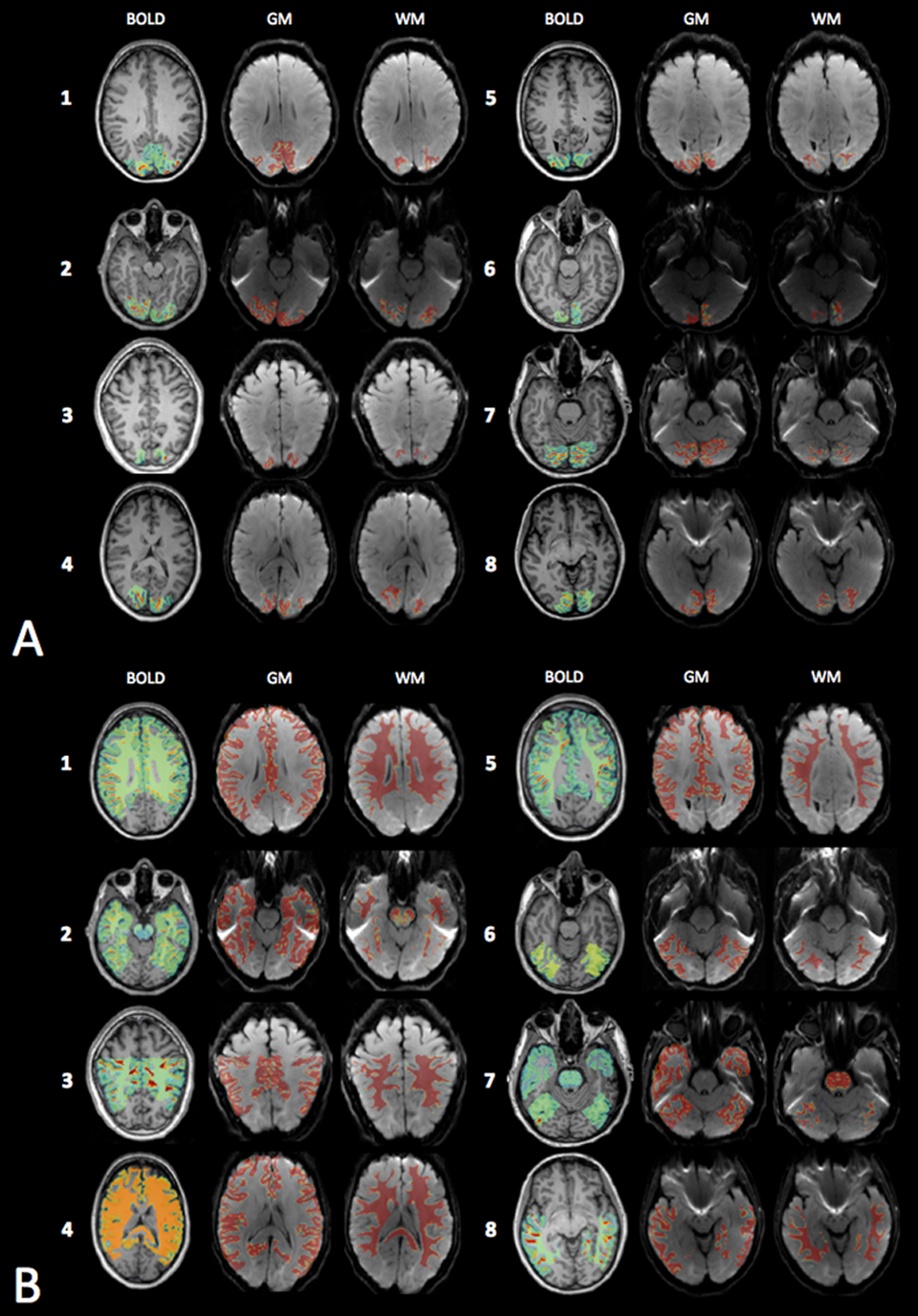

Supplement: S1 Fig — (A) Region of interest of the visual brain, as obtained by thresholding the t-map of the BOLD experiment, further segmented in gray and white matter, and coregistered to the b0 map of the IVIM sequence, and (B) of the non-visual brain, respectively. (TIF) [file pone.0117706.s001.tif]

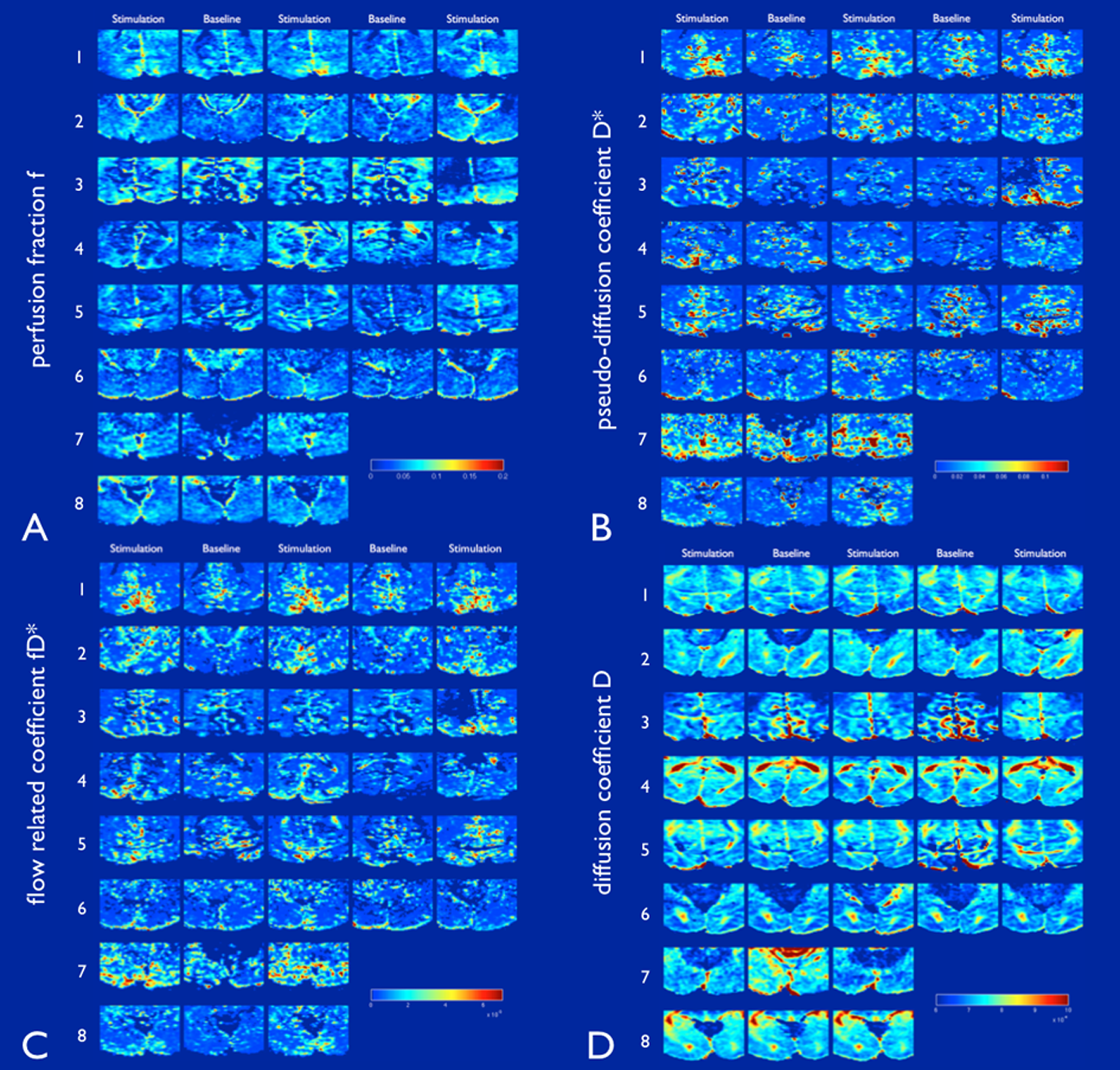

Supplement: S2 Fig — (A) Maps of the perfusion fraction f, (B) pseudo-diffusion coefficient D*, (C) flow related coefficient fD*, and (D) diffusion coefficient D, in all 5 (respectively 3) consecutive measurements for all 8 volunteers, during visual stimulation and rest (baseline). (TIF) [file pone.0117706.s002.tif]

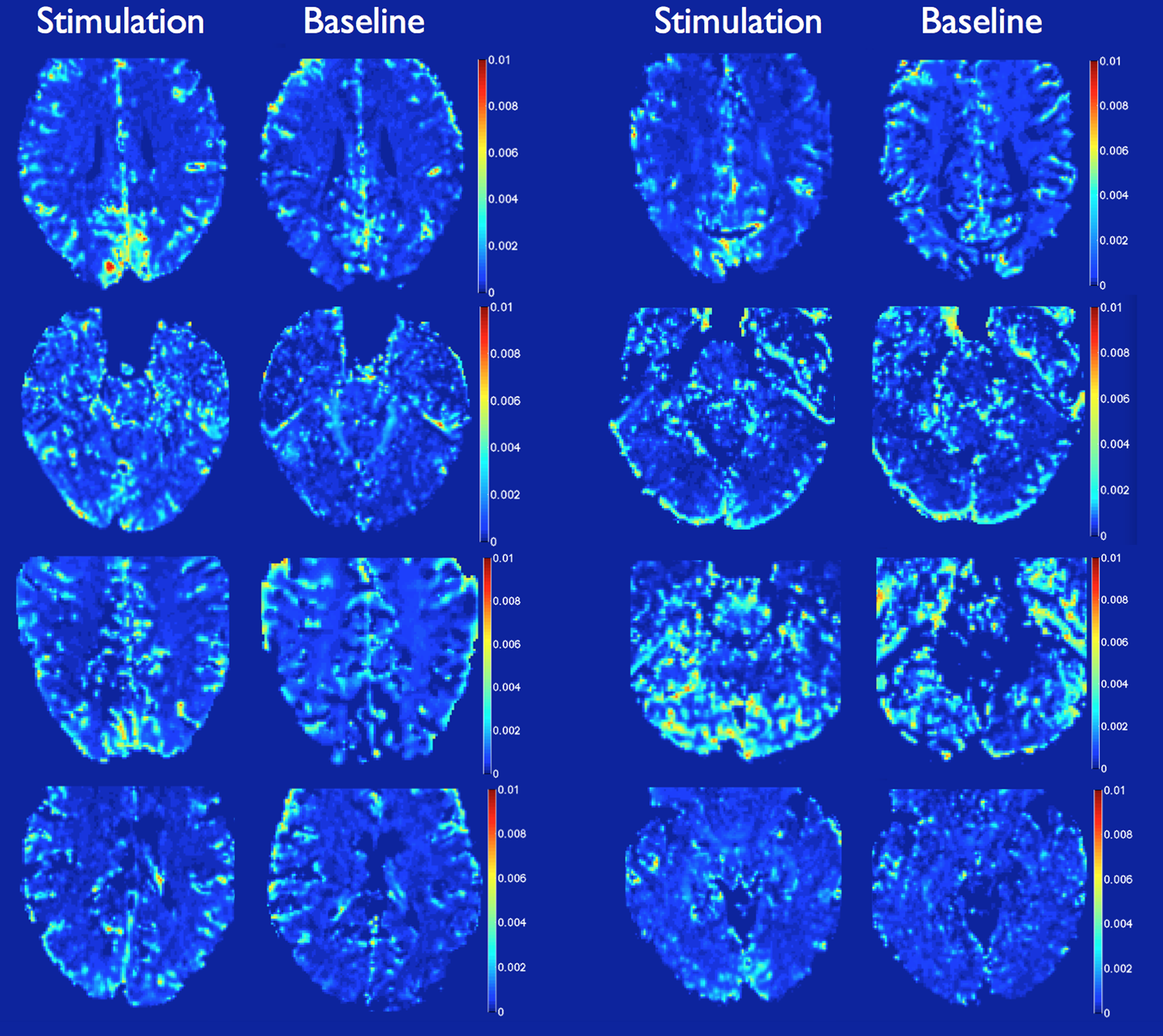

Supplement: S3 Fig — Maps of the blood flow related IVIM parameter fD*, in 8 volunteers, as obtained by averaging the maps obtained under visual stimulation and baseline. Scale of the colorbar: 10-3 mm2·s-1. (TIF) [file pone.0117706.s003.tif]
